# Global distribution, antimicrobial resistance, and virulence factors of *Staphylococcus epidermidis* revealed through population genomics

**DOI:** 10.1186/s12864-026-12922-5

**Published:** 2026-05-14

**Authors:** Yan Yang, Jian-Hua Liu, Cong-Ran Li, Yuan-Biao Guo, Xue Li

**Affiliations:** 1 Medical Research Center, the Affiliated Hospital of Southwest Jiaotong University, Third People’s Hospital of Chengdu, Chengdu, Sichuan 610031 China; 2 Department of Respiratory Medicine, the First Affiliated Hospital of Hebei North University, Zhangjiakou, Hebei 075000 China; 3 Institute of Medicinal Biotechnology, Chinese Academy of Medical Sciences & Peking Union Medical College, Beijing, 100050 China; 4 Research Center for Respiratory Medicine of Hebei North University, Zhangjiakou, Hebei 075000 China

**Keywords:** *Staphylococcus epidermidis*, Pan-genome, Phylogroup, Recombination rate, Blood infection

## Abstract

**Background:**

*Staphylococcus epidermidis*, typically regarded as a harmless commensal, has become one of the major causes of nosocomial infections, including ocular, skin, medical device-associated and bloodstream infections. Therefore, we analyze its population structure through genomic analysis integrated with metadata.

**Results:**

We performed whole-genome sequencing-based population genomic analyses by integrating 1742 publicly available *S. epidermidis* genomes (accessed by August 2025) with 94 newly sequenced isolates. Our analyses revealed that *S. epidermidis* represents a species complex composed of four phylogenetic lineages (phylogroups 1-4) with diverse clonal backgrounds and a broad global distribution. The species harbors an open pan-genome and demonstrates a strong capacity to acquire novel genetic traits through mobile genetic elements. Extensive antimicrobial resistance and substantial virulence potential were observed across lineages. Notably, phylogroup 1, dominated by ST 2, exhibited a 97.8% detection rate of the methicillin resistance gene *mecA*, likely driven by clonal expansion and horizontal gene transfer, identifying it as a high-risk lineage. The analysis of enriched genes in blood-derived strains showed that the adaptability of *S. epidermidis* in bloodstream-associated environments is controlled by multiple genes, involving antimicrobial resistance, cell wall remodeling, environmental adaptation, and core metabolism.

**Conclusions:**

This study provides a comprehensive population genomic framework for *S. epidermidis*, elucidating its population structure, genomic diversity, antimicrobial resistance, and virulence-associated genetic features. These findings offer valuable insights into the evolutionary dynamics and pathogenic potential of *S. epidermidis* and provide an important genomic resource to inform infection control strategies and clinical management.

**Supplementary Information:**

The online version contains supplementary material available at 10.1186/s12864-026-12922-5.

## Background

Coagulase-negative *Staphylococci* (CoNS) are a group of bacteria commonly found on human skin and mucous membranes. Historically, they have been regarded as low-virulence microorganisms or mere culture contaminants, owing to their widespread presence throughout the human body, including skin and nasopharynx [1]. Currently, they are increasingly recognized as significant pathogens in clinical settings due to their high infection rates and the complexity of the treatments required [2–4]. *Staphylococcus epidermidis*, a well-known coagulase-negative staphylococcus [5] typically regarded as a harmless commensal, has emerged as a major cause of nosocomial infections, including ocular, skin, medical device-associated and bloodstream infection [6]. Its pathogenic potential is largely attributed to its capacity to form biofilms, harbor antibiotic resistance determinants, and acquire mobile genetic elements, which collectively facilitate persistence and evasion of host defenses [7]. In addition to its role as an opportunistic pathogen, *S. epidermidis* also exhibits beneficial interactions within the microbial community. Moreover, recent research demonstrates that *S. epidermidis* fermentation broth can inhibit *Staphylococcus aureus* [8], emphasizing the significance of elucidating the genetic background of *S. epidermidis*.

To better understand the genetic diversity and population structure of *S. epidermidis*, several studies have used multilocus sequence typing (MLST) to investigate the relationships among isolates. These studies defined the broad population structure of *S. epidermidis* and identified multiple clonal lineages and genetic clusters, reflecting its adaptation to diverse ecological niches and lifestyles [9, 10]. Recent advances in high-throughput sequencing have provided unprecedented insights into the genomic architecture of *S. epidermidis*. Conlan et al. [11] analyzed 99 genomes and revealed substantial differences between skin commensal and hospital infection-associated isolates, offering important insights into the population structure and pan-genome of *S. epidermidis*. Lee et al. [12] further expanded this view by describing the genomic landscape of antibiotic resistance in this species on a larger scale. Moreover, Meric et al. [13] showed that although homologous recombination between the core genomes of *Staphylococcus aureus* and *S. epidermidis* is limited, mobile genetic elements related to antimicrobial resistance and virulence are frequently exchanged, highlighting the genomic plasticity and interspecies recombination boundaries of these two opportunistic pathogens. While previous studies have provided important insights into the population structure and genomic features of *S. epidermidis*, its global population structure and pan-genome remain incompletely resolved, partly because expanding genomic datasets continue to reveal additional lineage diversity and genetic variation.

To address this knowledge gap, we sequenced 94 non-duplicated clinical isolates of *S. epidermidis* and integrated 1,742 publicly available genomes, resulting in a comprehensive dataset of 1,836 genomes for pan-genome and comparative genomic analyses. Our aim was to thoroughly examine the genomic architecture of this extensive *S. epidermidis* collection, characterize genomic diversity across phylogroups, and integrate analyses of the core and accessory genomes, antibiotic resistance and virulence repertoires, and blood-origin-specific genes. This integrative framework provides a deep understanding of the genomic foundations underlying adaptation, pathogenicity, and antimicrobial resistance in this clinically important bacterium.

## Results

### Sequencing of 94 clinical *S. epidermidis* isolates uncovered highly diverse isolation sources

We sequenced 94 non-duplicated clinical isolates of *S. epidermidis* from the hospital in Hebei, China (Table S1). The 94 isolates were recovered from various types of clinical samples, including blood, secretion, urine, pleural effusion and drainage fluid. This distribution indicates that the isolates originated from a wide range of infection sites, and the pathogen can cause diverse types of infections across multiple body sites. The 94 isolates were assigned to 44 distinct sequence types (STs). Ten isolates could not be assigned an ST: nine due to novel allelic profiles and one due to deletion of the *arcC* gene. Among all STs, ST59 was the most common type (*n* = 11, 11.7%), followed by ST5 (*n* = 9), ST210 (*n* = 7), and ST235 (*n* = 4). In our collection, half of the strains (*n* = 47, 50.0%) belong to ST groups consisting of only one or two isolates and therefore did not exhibit intra-hospital transmission (Table S1).

### Global genomic diversity of *S. epidermidis*

**Fig. 1:**
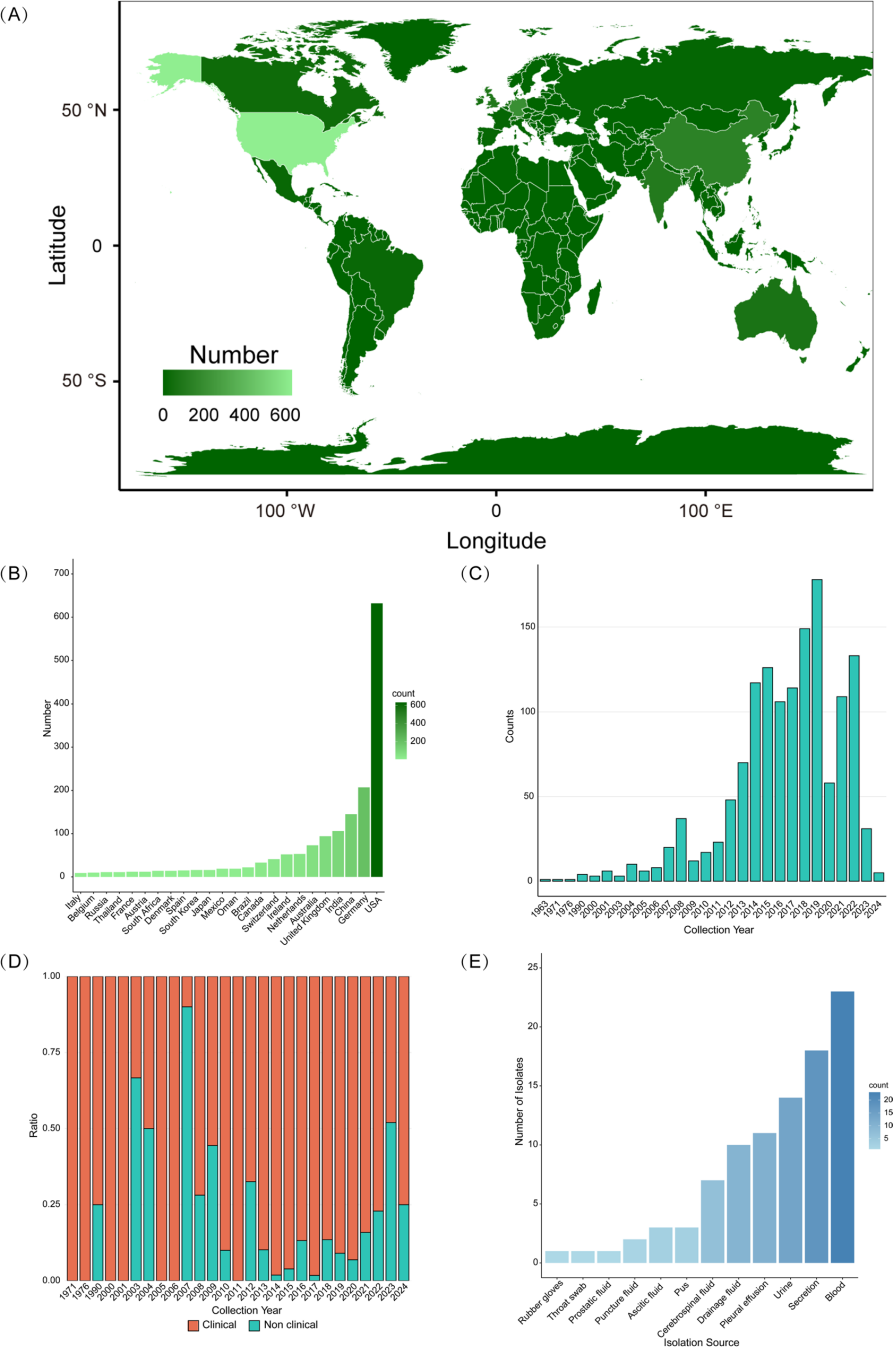
Overview of the dataset assembled in this study. **A** Geographic distribution of 1,836 *S. epidermidis* strains. The color intensity of each country corresponds to the number of strains isolated. **B** Bar chart showing the countries with the highest numbers of strains. **C** The bar chart showing the number of strains collected each year. **D** Source distribution across sample collection years. **E** Distribution of isolation sources among 94 *S. epidermidis* strains

We retrieved publicly available genomes annotated as *Staphylococcus epidermidis* from the RefSeq database (accessed August 2025). Genomes derived from metagenome-assembled genomes (MAGs) and those associated with large multi-isolate projects were excluded. In total, we identified 1,742 unique *S. epidermidis* BioSample accessions. These, together with the 94 genomes sequenced in this study, yielded a final dataset of 1,836 genomes (Table S1 and S2). Among the 1,836 isolates, 1,687 had specified geographic information and were recovered from 42 countries across six continents (Fig. 1A), including North America (*n* = 684), Europe (*n* = 555), Asia (*n* = 317), Oceania (*n* = 73), South America (*n* = 22), and Africa (*n* = 36). The countries contributing the largest numbers of isolates were the USA (*n* = 632), Germany (*n* = 207), China (*n* = 145), India (*n* = 106), the United Kingdom (*n* = 94), Australia (*n* = 73), and the Netherlands (*n* = 53) (Fig. 1B). Among the 1,836 strains, the earliest isolates date back to the 1960s, although the majority were collected between 2012 and 2022 (Fig. 1C). Overall, these isolates were primarily obtained from clinical samples; however, in certain years, the proportion recovered from non-clinical environments even exceeded that from clinical sources (Fig. 1D). In this study, all 94 *S. epidermidis* isolates were collected from hospitalized patients. Among them, blood-derived isolates were the most common (*n* = 23, 24.0%), followed by secretion (*n* = 18, 19.1%), urine (*n* = 14, 14.9%), pleural effusion (*n* = 11, 11.7%) and drainage fluid (*n* = 10, 10.6%) (Fig. 1E). The predominance of blood-derived isolates indicates a close association with bloodstream infections. However, it should be noted that isolates obtained from these non-blood sites cannot be definitively considered causative pathogens, as they may represent colonizing or contaminating strains rather than true infectious agents. Nevertheless, these observations still highlight the ability of *S. epidermidis* to colonize diverse body sites and its versatility in opportunistic infections.

### *S. epidermidis* exhibits a remarkable clonal diversity comprising 237 STs and four phylogroups

**Fig. 2:**
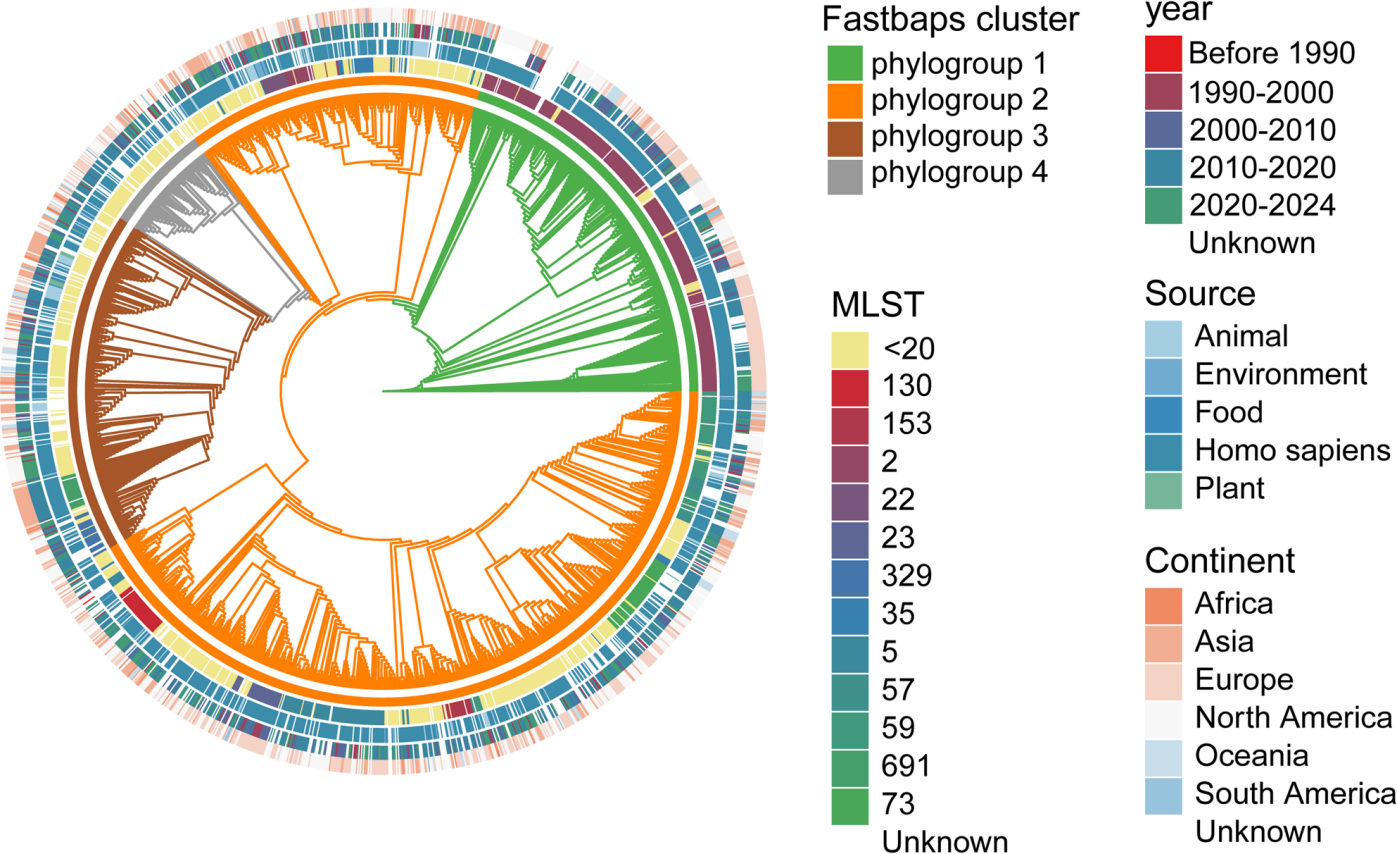
Circular phylogenomic tree of *S. epidermidis* with isolates information. 1,742 genomes of *S. epidermidis* from RefSeq database (accessed by August 2025) and the 94 genomes of *S. epidermidis* sequenced in this study were included. The five outer rings depict Fastbaps cluster (corresponding to phylogroups), MLST type, ecological source, the year of sampling, and continent. Sequence types comprising 20 or more isolates are shown. The phylogenetic tree was reconstructed by IQ-TREE basing on concatenated SNPs from core genes present in at least 95% of the genomes, applying the GTR + GAMMA model with ascertainment bias correction and 1,000 bootstrap replicates

Population structure analysis of the core genome alignment using Fastbaps identified four distinct sequence clusters (phylogroup 1-4) (Fig. 2). The majority of the *S. epidermidis* isolates (*n* = 1,045, 56.9%) belonged to phylogroup 2, followed by 368 isolates (20.0%) in phylogroup 1, 322 isolates (17.5%) in phylogroup 3, 101 isolates (5.5%) in phylogroup 4 (Fig. 2). When the Fastbaps results were mapped onto the phylogenetic tree, phylogroup 2 was found to span two clades (Fig. 2). This indicates that Fastbaps clusters capture genetic similarity rather than strictly follow evolutionary lineage in here, likely influenced by recombination or horizontal gene transfer events. A total of 1,836 *S. epidermidis* isolates were assigned to 237 STs. Among these, 171 isolates could not be assigned an ST because they harbored novel alleles and/or novel allelic profiles (Fig. 2 and Table S2). Phylogroup 1, 2, 3, and 4 comprised 7, 135, 78, and 19 STs, respectively. ST2 was the most prevalent type, encompassing 357 isolates, followed by ST5 (109 isolates), ST59 (86 isolates), and ST73 (65 isolates) (Fig. 2). Notably, ST2 was distributed across two distinct phylogroups (332 isolates in phylogroup 1 and 25 isolates in phylogroup 2). Because MLST is based on only seven housekeeping gene loci and therefore does not fully capture evolutionary relationships across the whole genome, this distribution suggests that ST2 may have experienced independent evolutionary events or horizontal gene transfer, resulting in divergent genomic backgrounds [14, 15]. Furthermore, 104 STs were represented by a single isolate, while only 37 STs contained ten or more isolates.

STs were classified as international or intercontinental based on their presence in isolates from at least three continents, following the criteria described by Feng, et al. [16]. As a result, 40 STs were identified as intercontinental. Notably, ST2, ST6, ST35, and ST59 exhibited truly global distributions, each occurring across all six continents. Specifically, ST2 was identified in 22 countries; ST6 appeared in 7 countries despite being represented by only 10 isolates in the dataset; ST35 was detected in 12 countries; and ST59 was found in 19 countries. Together, these findings indicate that ST2, ST6, ST35, and ST59 are globally disseminating lineages across all six continents (Table S1 and S2).

### *S. epidermidis* has an open genome revealed by pan-genome analysis

**Fig. 3:**
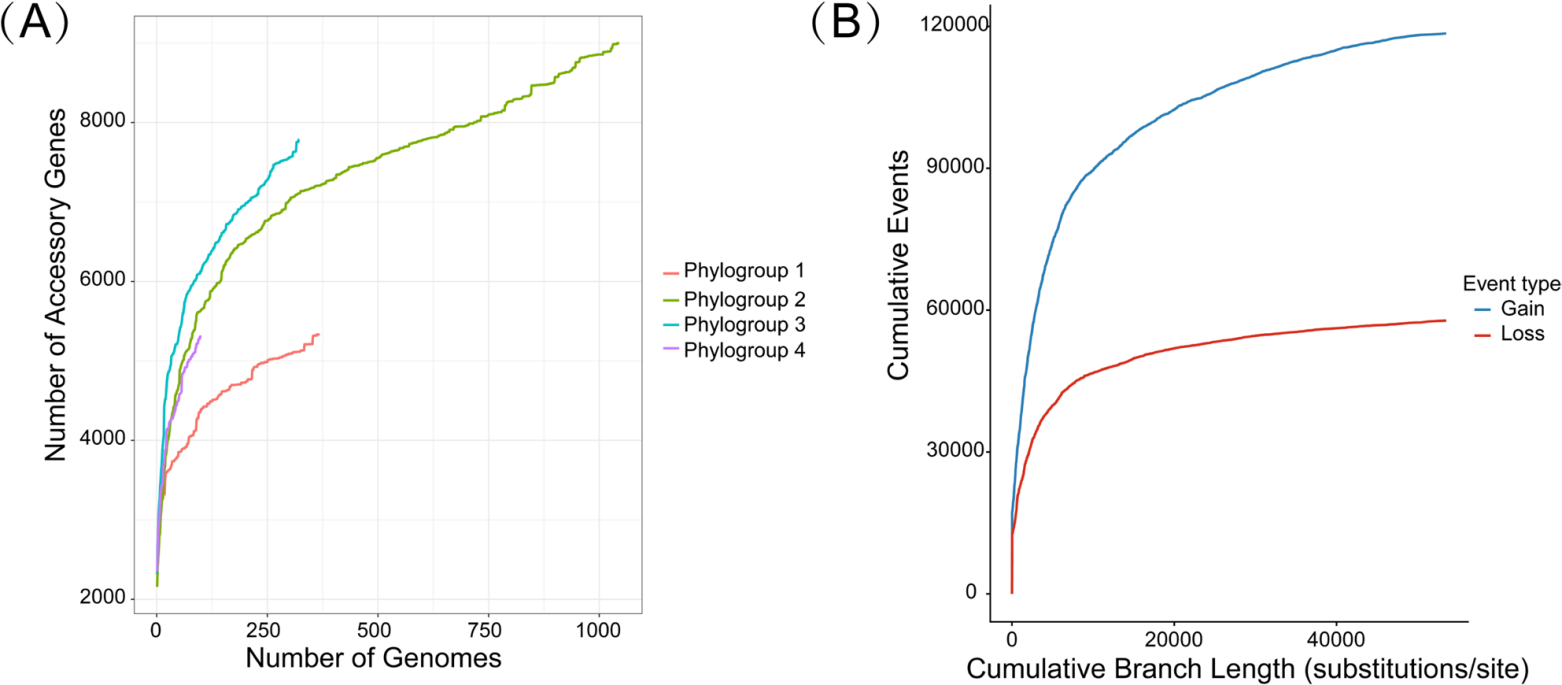
The *S. epidermidis* genome is open, as revealed by pan-genome analysis. **A** Pan-genome accumulation curves showing the number of accessory genes across the four phylogroups. The pan-genome matrix was generated using Panaroo. **B** Cumulative numbers of gene gain and loss events plotted against the cumulative core branch length

Pan-genome analysis of the global collection of 1,836 *S. epidermidis* strains revealed a highly diverse genomic landscape, comprising 11,233 non-redundant genes across all strains. A total of 1,846 genes were identified as core genes, present in at least 95% of strains, these genes constitute the conserved core genome of *S. epidermidis*. The pan-genome accumulation curve of the analyzed strain indicated an open pan-genome, with the total number of genes continuing to increase as additional genomes were included (Fig. 3A). This suggests that *S. epidermidis* harbors extensive genomic diversity, and new genes-particularly accessory genes-are likely to be discovered with the sequencing of more isolates. We also performed analysis using a saturation curve of unique gene clusters under the Heaps’ law model [17], which yielded a α value of 0.187. Since α value of < 1 indicates that new genes continue to be discovered as additional genomes are sequenced (reflecting a high level of genetic diversity within the species), the observed α value of 0.187 confirms that *S. epidermidis* has an open pan-genome.

We then assessed how the cumulative number of gene gain and loss events varied with cumulative core branch length. The results showed that these events increased with branch length, indicating a positive correlation between genome evolution and gene content diversification (Fig. 3B). After normalization by branch length, gene gain occurred at nearly twice the rate of gene loss after normalization by branch length (2.21 vs. 1.08 events per unit branch length), indicating a pronounced asymmetry in gene turnover (Fig. 3B). This pattern suggests that gene gain and loss events in *S. epidermidis* are largely driven by recent mobile genetic elements that have not yet spread widely across the population.

### Variation in mutation, recombination and genomic diversity across phylogroups

**Fig. 4:**
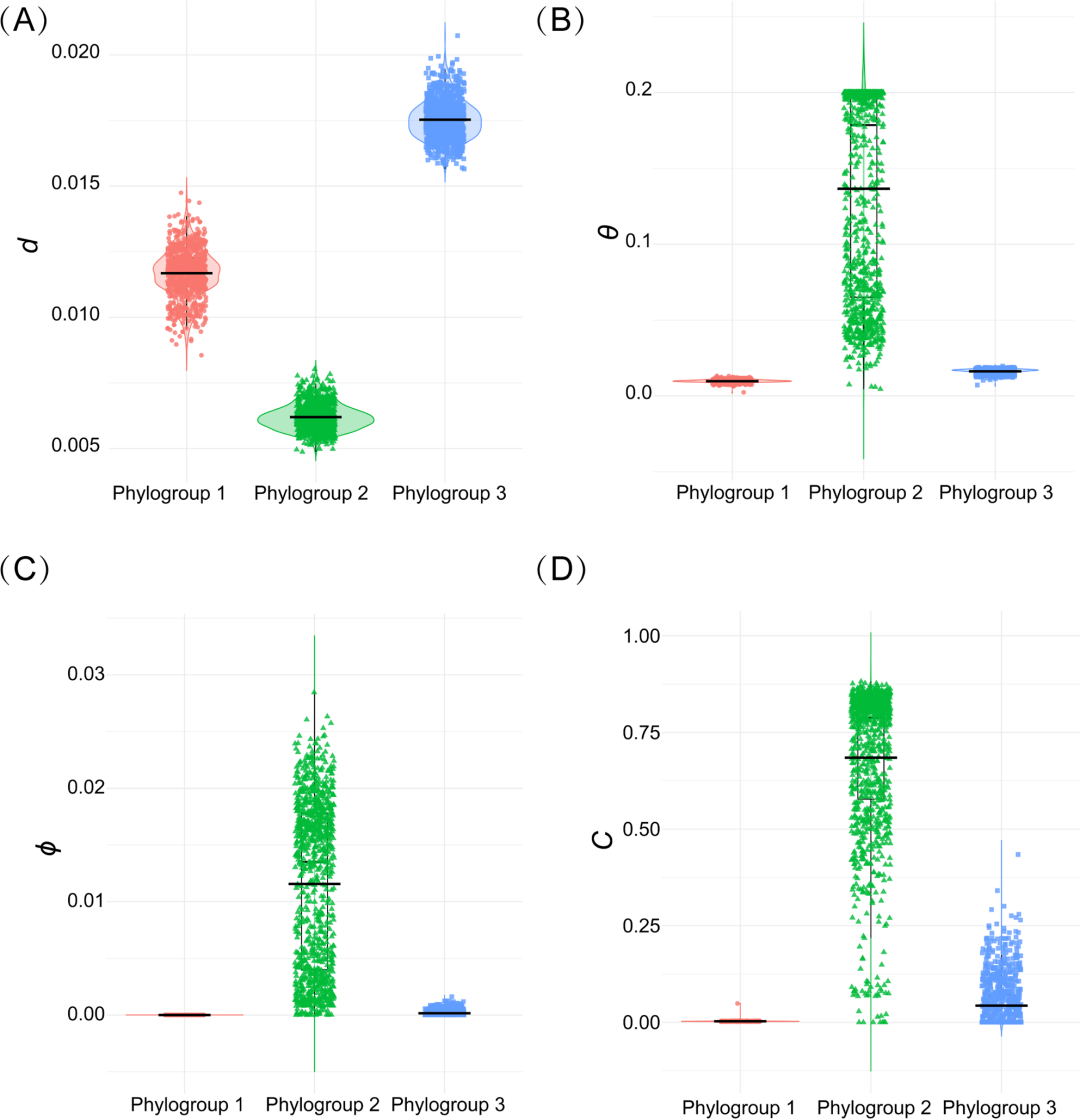
Mcorr-based comparison of evolutionary parameters across three phylogroups of *S. epidermidis.* Violin plots show the distributions of (**A**) overall nucleotide diversity (*d*), reflecting variation generated by both mutation and homologous recombination; **B** mutation-driven nucleotide diversity (θ); **C** recombination-associated diversity (ϕ); and (**D**) recombination coverage (c) across the three phylogroups

We next sought to determine whether recombination or mutation plays the predominant role in driving genome evolution within phylogroup 1, 2, and 3. These three phylogroups were selected because they contain the largest number of genomes. Using the core genome alignment and 1,000 bootstrap replicates as input, we employed Mcorr to estimate key recombination parameters [18, 19]. Mcorr analysis revealed marked heterogeneity among the three phylogroups in core-genome diversity and homologous recombination. In the full dataset of 1,836 genomes, diversity (*d*) was lowest in Phylogroup 2, intermediate in Phylogroup 1, and highest in Phylogroup 3, indicating that Phylogroup 3 harbors the most diverged core-genome lineages whereas Phylogroup 2 is comparatively homogeneous (Fig. 4A). In contrast, the Mcorr-derived mutational divergence (*θ*), recombinational divergence (*ϕ*), and recombination coverage (*c*) were all highest in Phylogroup 2 (Fig. 4). Although Phylogroup 1 showed intermediate *d* values, it consistently exhibited a low estimate of recombinational divergence (*ϕ*) and recombination coverage (c), consistent with a more clonal population structure (Fig. 4). This interpretation is supported by its MLST composition, as most Phylogroup 1 isolates belonged to ST2 (332/368) (Fig. 2 and Table S2).

To assess whether these patterns were influenced by uneven geographic sampling, we repeated the analysis using a country-balanced dataset (Table S3 and Figure S1). However, after balancing the number of isolates across countries, the relative magnitude of these parameters shifted, with stronger recombination-related signals observed in Phylogroup 3 (Figure S1). This suggests that Mcorr estimates, particularly those related to recombination, are sensitive to sampling structure and geographic representation. Nevertheless, the overall phylogroup-level heterogeneity was reproducible across analyses: Phylogroup 1 was characterized by an ST2-dominated, weakly recombining structure, Phylogroup 2 consistently showed low core-genome diversity, and Phylogroup 3 harbored the most divergent core genomes. Overall, these findings highlight the complex interplay between genetic diversity, recombination dynamics, and population structure in shaping the evolutionary trajectories of *S. epidermidis* across different phylogroups.

### Resistance and virulence genes of the *S. epidermidis* genomes

**Fig. 5:**
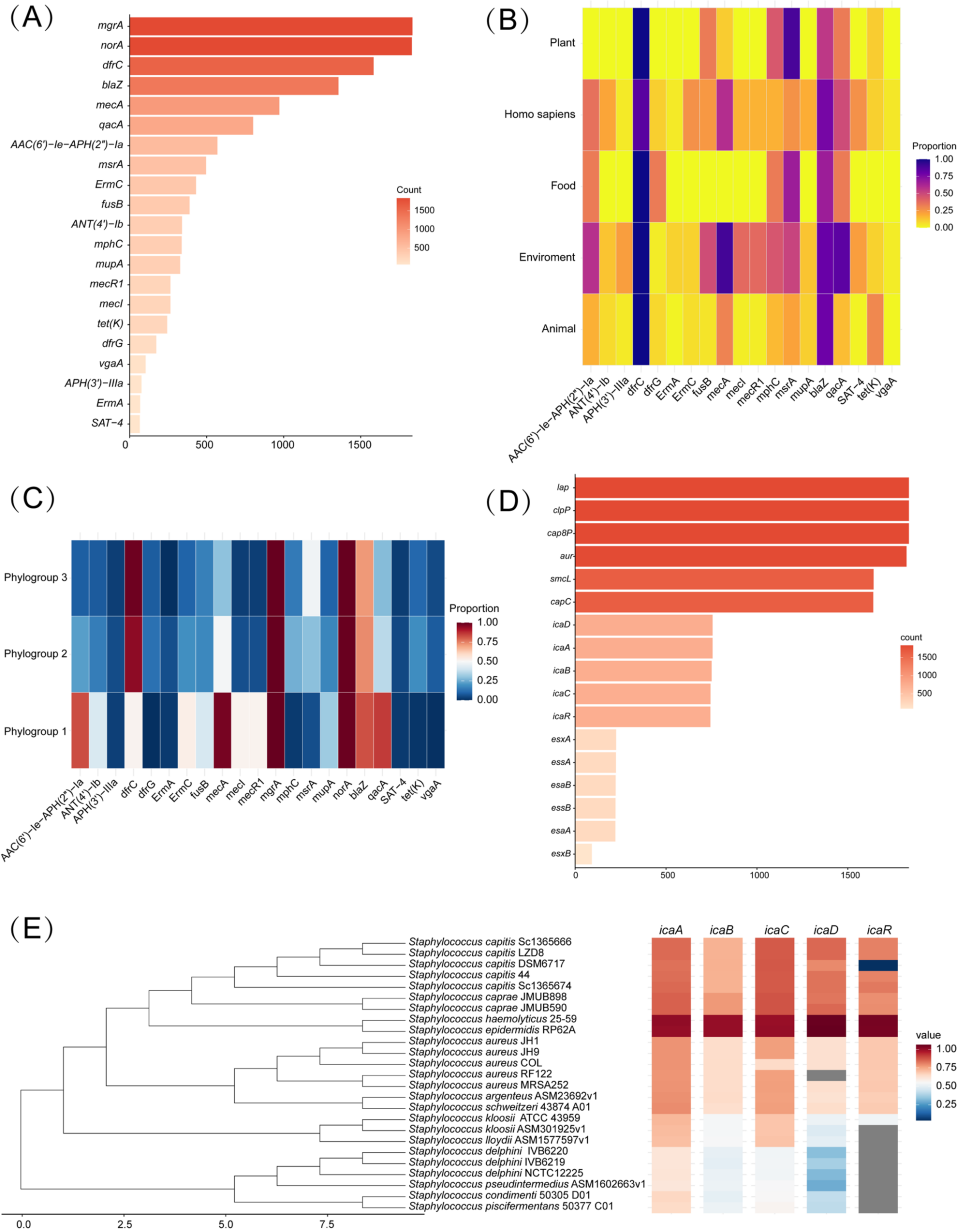
Resistance and virulence genes of the *S. epidermidis* genomes. **A** Bar plot showing the distribution of resistance genes identified in 1,836 *S. epidermidis* isolates. Only genes presenting more than 50 times are displayed. **B** Distribution of antimicrobial resistance genes across different sources. **C** Distribution of antimicrobial resistance genes among the three phylogenetic clusters. **D** Bar plot depicting the distribution of virulence genes in 1,836 *S. epidermidis* isolates. Only genes presenting more than 90 times are displayed. **E** Homology analysis of the *icaABCDR* operon across *Staphylococcus* species. Gray shading indicates gene absences

Among the 1836 *S. epidermidis* isolates, numerous antibiotic resistance genes were detected, spanning multiple classes including β-lactams, aminoglycosides, macrolides, quinolones, sulfonamides, and disinfectant resistance genes (Fig. 5A). The genes *mgrA* (1,836 isolates) and *norA* (1,832 isolates) were nearly ubiquitous, indicating that these two genes are highly conserved in the *S. epidermidis* population. Other highly prevalent genes included *blaZ* (1,356 isolates), *mecA* (972 isolates), *mecI* (265 isolates), and *mecR1* (265 isolates), indicating widespread resistance to β-lactam antibiotics, particularly penicillins and methicillin. Approximately half of the isolates carrying *mecA*, consistent with the presence of methicillin-resistant *S. epidermidis* (MRSE) reported in previous clinical studies [15, 20]. Aminoglycoside resistance genes such as *AAC(6’)-Ie-APH(2’’)-Ia* (570 isolates), *ANT(4’)-Ib* (340 isolates) and *APH(3’)-IIIa* (77 isolates), and macrolide resistance genes including *msrA* (496 isolates), *ermC* (431 isolates), and the *vgaA/B* family were found in some of the isolates, indicating the presence of multidrug-resistant strains in the population. The distribution of resistance genes in *S. epidermidis* was broader in environmental sources, followed by human-associated sources (Fig. 5B). These observations suggest that the environment could represent a potential source of resistance genes, although the relatively small number of environmental isolates limits the strength of this inference. Notably, methicillin resistance associated genes (including *mecA*, *mecI*, and *mecR1*) were particularly prevalent in environmental isolates. Animal-derived samples also carried antibiotic resistance genes. Importantly, the *tet(K)* gene was detected at a notably higher frequency in animal samples (29.4%, 40/136) compared with human-derived samples (7.2%, 132/1,836), suggesting that this gene may be more prevalent in animals than in other sources. Overall, these findings highlight distinct patterns of antimicrobial resistance gene distribution across different sources, suggesting that the environment and animals may serve as potential reservoirs for specific resistance genes, though further investigation is needed to confirm these associations. Next, we aimed to determine the distribution of antimicrobial resistance genes within phylogroup 1, 2, and 3. The analysis revealed that phylogroup 1 exhibited a relatively higher abundance of resistance genes compared with the other two phylogroups (Fig. 5C). This suggests that phylogroup 1 may be subject to stronger selective pressures or more frequent horizontal gene transfer, highlighting its potential clinical significance in terms of multidrug resistance.

Using a relatively relaxed 70% sequence identity threshold in the VFDB (Virulence Factor Database) database, we identified virulence-associated genes in 1,836 *S. epidermidis* isolates (Fig. 5D). Genes such as *clpP* (1836), *cap8P* (1,836), *lap* (1,836), *capC* (1,640), *aur* (1,823), and *smcL* (1,642) were present in nearly all isolates. The *ica* locus, mediating polysaccharide intercellular adhesin (PIA) production, was detected in a subset of isolates: *icaD* (756), *icaA* (756), *icaB* (751), *icaC* (745), and *icaR* (744), indicating variation in biofilm-forming potential. Type VII Secretion System (T7SS) components were also observed in multiple isolates, including *esxA* (225), *essA* (223), *esaB* (223), *essB* (222), *esaA* (221), and *esxB* (92). Using the *S. epidermidis* RP62A (NC_002976) *icaABCDR* operon (SERP2292-6) as a reference, we further examined the amino acid sequence similarity of homologous *ica* genes across other *Staphylococcus* species. Comparative analysis revealed that the sequence identity of these *ica* proteins ranged from 60% to 90%, indicating substantial divergence at the protein level (Fig. 5E). This divergence likely reflects species-specific evolutionary paths of the *ica* locus, which may contribute to functional differences in PIA biosynthesis and biofilm formation. These observations warrant further investigation in the future.

### Enriched genes in blood-derived *S. epidermidis* isolates

To investigate potential genetic features associated with bloodstream infections, we compared the accessory gene profiles of blood and non-blood sources isolates using the Panaroo-generated presence/absence matrix. Comparative genomic analysis identified a set of differentially enriched genes associated with antimicrobial resistance, cell wall remodeling, environmental adaptation, and core metabolism (Table 1). Resistance-associated genes, including *mecA*, *mecR1*, *blaZ*, and *aacA*, indicated divergence in the potential to withstand β-lactam and aminoglycoside exposure. Genes involved in cell wall turnover and biofilm-related processes, such as *atl* and *ssaA*, suggested differences in adhesion, autolysis, and persistence capacity. In addition, enrichment of *hmuU*, *copZ*, *cadA*, and *nmtR* pointed to variation in heme acquisition, metal ion homeostasis, and stress adaptation. Several genes involved in nucleotide biosynthesis, DNA replication/repair, and central metabolism, including *thyA*, *folA*, *dnaB*, *recD*, *gloB*, *hisB*, *hisI*, and *mvaS*, were also differentially represented, indicating broader physiological divergence between the two isolate groups. Collectively, these findings suggest that blood- and non-blood-derived isolates differ across multiple functional categories rather than in a single pathogenicity-associated pathway.

**Table 1:** Significantly enriched genes in blood-derived versus non-blood-derived isolates identified by multivariable analysis adjusting for phylogroup and country

Gene	Function	*p*.value	OR (95% CI)	p_adj
Antibiotic resistance and disinfectant tolerance				
* mecA*	Methicillin and β-lactam resistance	5.68E-07	6.58 (3.14–13.77)	7.20E-05
* mecR1*	Regulate *mecA* expression	1.42E-05	4.46 (2.27–8.76)	9.01E-04
* aacA*	Aminoglycoside acetyltransferase	6.46E-05	3.98 (2.02–7.85)	3.41E-03
* qacR*	Regulates the expression of qacA/B efflux pumps	1.39E-03	2.90 (1.51–5.56)	2.86E-02
* blaZ*	β-lactamase	2.08E-03	2.59 (1.41–4.73)	3.84E-02
DNA replication, recombination and repair				
* dnaB*	DNA helicase	6.99E-06	4.08 (2.21–7.53)	4.93E-04
* recD*	Subunit of the RecBCD complex	7.19E-04	11.99 (2.84–50.57)	1.80E-02
Metabolism and biosynthesis				
* thyA*	Thymidylate synthase	2.34E-10	11.42 (5.38–24.25)	1.48E-07
* folA*	Dihydrofolate reductase	2.03E-08	13.19 (5.36–32.48)	3.87E-06
* mvaS*	HMG-CoA synthase	3.33E-04	3.09 (1.67–5.73)	1.15E-02
* nmoA*	N-formylmethionine oxidase	1.06E-03	3.76 (1.70–8.31)	2.44E-02
* hisB*	Imidazoleglycerol-phosphate dehydratase	1.18E-03	3.03 (1.55–5.93)	2.54E-02
* hisI*	Histidinol-phosphate phosphatase	2.30E-03	4.21 (1.67–10.61)	4.13E-02
* gloB*	Glyoxalase II	7.16E-04	3.48 (1.69–7.17)	1.80E-02
Cell wall, virulence and adhesion				
* atl*	Autolysin	2.75E-06	6.70 (3.02–14.84)	2.49E-04
* ssaA*	Staphylococcal surface protein	8.47E-04	2.78 (1.52–5.06)	2.09E-02
* pre*	Proprotein convertase	1.90E-03	3.37 (1.56–7.24)	3.58E-02
* larC*	Lipoteichoic acid synthesis and modification	2.23E-03	3.06 (1.49–6.27)	4.04E-02
* essD*	Type VII secretion system	2.79E-03	32.31 (3.31-315.28)	4.57E-02
Metal ion homeostasis and stress resistance				
* hmuU*	Hemin/iron transport permease	3.72E-04	4.00 (1.86–8.59)	1.24E-02
* cadA*	Cadmium/zinc resistance transporter	1.25E-03	4.76 (1.84–12.26)	2.61E-02
* copZ*	Copper chaperone protein	1.66E-03	4.50 (1.76–11.49)	3.25E-02
* nmtR*	Nickel/cobalt-sensing transcriptional regulator	2.94E-03	3.24 (1.49–7.03)	4.74E-02
Transcriptional regulation				
* lrpC*	Leucine-responsive transcriptional regulator	2.05E-03	2.99 (1.49–5.98)	3.82E-02

*OR* Odds Ratio, *CI* Confidence interval, *p_adj*  Adjusted *p*-value

## Discussion

The increasing prevalence of multidrug-resistant *S. epidermidis* presents a substantial challenge for clinical treatment [21]. In this study, we characterized the population structure of *S. epidermidis* and demonstrate that it constitutes a species complex composed of multiple phylogenetic lineages with highly diverse clonal backgrounds and a broad global distribution. Our analysis shows that *S. epidermidis* harbors an open pan-genome and exhibits a strong capacity to acquire novel genetic traits through mobile genetic elements. Moreover, the species displays extensive antimicrobial resistance, notable virulence potential, and pronounced lineage-specific adaptations. Collectively, these findings provide new insights into an understudied yet clinically significant pathogen and offer valuable information to inform infection control strategies and improve clinical management.

The core genome-based population structure analysis revealed four major phylogroups, indicating substantial genomic diversity within the global *S. epidermidis* population. Although 237 sequence types were identified, a small number of dominant lineages accounted for most isolates, while over 100 STs were represented by a single strain, reflecting extensive diversification with a vast pool of rare genotypes. Notably, 40 STs exhibited intercontinental distribution, ST2, ST6, ST35 and ST59 were detected across all inhabited continents, indicating successful global dissemination. Together, these findings show that the worldwide *S. epidermidis* population comprises both highly successful epidemic lineages with broad geographic spread and numerous locally restricted or sporadic genotypes.

Consistent with earlier work showing that *S. epidermidis* harbors an open and highly dynamic pangenome [11], the continuously increasing pangenome curve and the Heaps’law ɑ value of 0.187 further confirm that new accessory genes will continue to emerge as additional isolates are sequenced. The large and expanding pangenome, along with the strong enrichment of recent gene gain and loss events on terminal branches, indicates that horizontal gene transfer plays a central role in generating lineage-specific diversity on short evolutionary timescales.

Our analysis revealed substantial differences among the three phylogroups in mutation-, recombination-, and diversity-related parameters. Across analyses, the most consistent pattern was that Phylogroup 2 showed the lowest core-genome diversity, whereas Phylogroup 3 harbored the most divergent core genomes. In contrast, Phylogroup 1 was characterized by a low recombination-related estimates and a strong predominance of ST2, supporting a comparatively clonal population structure. This suggests that the evolutionary success of Phylogroup 1 may be associated more with the emergence and subsequent clonal expansion of an ST2-associated lineage than by high levels of ongoing homologous recombination. Although Phylogroup 1 includes the ancestral sequence type of the previously described CC2 (clonal complex) [10], our results do not support a strict one-to-one correspondence between Phylogroup 1 and CC2. Rather, Phylogroup 1 appears to partially overlap with the broader ST2-centered lineage framework described in earlier studies. ST2 is the founder sequence type of CC2 and represents a predominant, geographically widespread invasive lineage of *S. epidermidis* [10, 14]. In hospital settings, ST2 has been repeatedly associated with amplification and diversification of *SCCmec* (staphylococcal cassette chromosome *mec*), carriage of IS256 and the *ica* operon, enhanced biofilm-forming capacity, and multidrug resistance, all of which likely promote persistence on abiotic surfaces and indwelling devices under intense antimicrobial selection [15, 22–24]. ST2’s evolution likely relied on vertical clonal expansion and the retention of beneficial mobile elements, potentially due to the presence of restriction-modification systems that limit homologous recombination [25]. These findings highlight the key role of clonal expansion and mobile genetic element retention in the evolutionary success of ST2, underscoring its adaptation to hospital environments and its capacity for persistence under selective antimicrobial pressure.

Our comprehensive genomic analysis of 1,836 *S. epidermidis* isolates highlights the species’ extensive antimicrobial resistance, virulence potential, and lineage-specific adaptations. MgrA, also known as NorR, is a multifaceted regulator of *norA*, *norB*, and *tet38* [26, 27]. Both *mgrA* and *norA* are nearly ubiquitous across the population, suggesting that these genes are conserved in *S. epidermidis* as they are in *Staphylococcus aureus* [28], indicating that these proteins play a crucial role in the bacterial physiological functions. *S. epidermidis* is considered a reservoir for methicillin resistance, capable of transferring this resistance to other species, including *S. aureus* [29, 30]. Among 1,836 *S. epidermidis* isolates, the detection rate of the methicillin resistance gene *mecA* was 53.0% (973/1,836), with phylogroup 1 showing a 97.8% detection rate (360/368). These further underscores the central role of phylogroup 1, dominated by ST2, in the spread of methicillin resistance, potentially due to its clonal expansion and horizontal gene transfer [14]. The presence of other prevalent resistance determinants, including aminoglycoside and macrolide resistance genes, highlights the emergence of multidrug-resistant strains in a subset of isolates.

Virulence profiling revealed that the *S. epidermidis* harbors highly conserved virulence determinants (e.g., *clpP*, *cap8P*, *lap*, *aur*) as well as loci associated with biofilm formation (*icaABCDR*) and the T7SS [31]. The *icaADBC* operon is responsible for the synthesis, modification, and export of the PIA, facilitating bacterial adhesion, accumulation, and the formation of mature biofilms, thereby constituting the central biofilm-associated virulence mechanism of *S. epidermidis* [32–34]. Comparative analysis of the *icaABCDR* operon across the *Staphylococcus* genus shows that the operon in *S. epidermidis* shares the highest homology with *S. haemolyticus*, while exhibiting lower homology with species that are more closely related to *S. epidermidis*, such as *S. caprae* and *S. capitis* [35]. This suggests that the conservation of the *icaABCDR* operon is not determined solely by phylogenetic relatedness but may instead reflect similar ecological pressures faced by *S. epidermidis* and *S. haemolyticus*, including adaptation to hospital environments or colonization of implanted medical device surfaces. Consistent with this ecological overlap, epidemiological data show that *S. epidermidis* and *S. haemolyticus* are also the most frequently detected CoNS species in clinical bloodstream infections [5].

Notably, the genes enriched in blood-derived isolates (Table 1) suggest that bloodstream-associated adaptation in *S. epidermidis* is polygenic and involves multiple functional modules rather than a single canonical virulence pathway. This interpretation is consistent with the current view that *S. epidermidis* is primarily an opportunistic pathogen whose clinical success depends largely on biofilm formation, protection from host defenses, and tolerance to antimicrobial exposure, rather than on an extensive repertoire of aggressive toxins [11, 36]. The enrichment of *mecA*, *mecR1*, *blaZ*, and *aacA* is therefore biologically plausible, as these genes are well-recognized determinants of β-lactam and aminoglycoside resistance and are common components of the *S. epidermidis* resistome, particularly among clinically associated lineages [12]. Likewise, the differential representation of *atl* and *ssaA* points to variation in cell-wall remodeling and biofilm-associated phenotypes. In *S. epidermidis*, the major autolysin AtlE promotes primary attachment to abiotic and plasma-protein-coated surfaces, while autolysis-mediated extracellular DNA release contributes to early biofilm development [37, 38]. These observations are consistent with the ecology of *S. epidermidis* bloodstream infection, which often originates from biofilm growth on indwelling medical devices [36]. The enrichment of *hmuU*, *copZ*, *cadA*, and *nmtR* further suggests adaptation to the metal-restricted and metal-stressed environment of blood. Although the specific role of HmuU in *S. epidermidis* remains to be experimentally validated, previous studies have shown that exposure to human blood induces iron-utilization pathways, and that iron-acquisition loci contribute to biofilm formation and survival within the host [39]. Finally, the differential representation of genes involved in folate and nucleotide metabolism, DNA replication/repair, and central metabolism suggests that bloodstream-associated isolates may also differ in broader physiological capacity. Collectively, these findings indicate that blood-derived isolates are characterized by a composite accessory-genome signature involving antimicrobial resistance, cell-envelope remodeling, nutrient acquisition, and stress adaptation, rather than a single bloodstream-specific pathogenicity determinant.

Several limitations should be considered when interpreting these findings. First, the dataset is geographically imbalanced, and although we performed country-balanced subsampling that also reduced ST overrepresentation, complete equalization of population structure was not possible, especially in Phylogroup 1 because of its intrinsic ST2 dominance. Second, recombination-related estimates derived from Mcorr are influenced by sampling structure and alignment strategy, and should therefore be interpreted comparatively rather than as absolute measures of recombination intensity. Third, environmental and animal isolates were less numerous than human-derived isolates, which limits the strength of ecological inference. Finally, batch effects, stemming from differences in sequencing platforms, depth, and analysis methods between on-site sequencing and publicly available databases, could still influence certain aspects of the data, despite efforts to minimize their impact.

In summary, our study reveals that distinct patterns of genome evolution differentiate the phylogroups of *S. epidermidis*. Our findings provide important insights into the genetic determinants for ecological versatility, virulence, and multidrug resistance features in the species. This knowledge will inform current efforts to control and manage diseases, including effective approaches to target specific *S. epidermidis* phylogroups, clones, or lineages.

## Conclusions

*Staphylococcus epidermidis* is a common human commensal and a leading cause of hospital-acquired infections, yet it is often considered genetically uniform. By integrating a large global genome collection with clinically collected isolates, we show that *S. epidermidis* is a species complex comprising distinct phylogroups with divergent evolutionary dynamics, antimicrobial resistance, and virulence potential. These findings offer valuable insights into the evolutionary dynamics and pathogenic potential of *S. epidermidis* and provide an important genomic resource to inform infection control strategies and clinical management.

## Materials and methods

### Bacterial strains

A total of 94 non-repetitive *S. epidermidis* between 2017 and 2024 were collected from The First Affiliated Hospital of Hebei North University, a Grade-III ClassA Hospitals in Hebei Province from north China and stored in the Chinese Academy of Medical Sciences Collection Center of Pathogen Microorganisms (CAMS-CCPM-AP).

### Whole genome sequencing

All of the 94 isolates were subjected to whole genome sequencing as performed on the Illumina NovaSeq 6000 platform (Illumina, San Diego, CA, USA) using a paired-end 150 bp (PE150) strategy at Beijing Novogene Bioinformatics Technology Co., Ltd. Specifically, the genomic DNA was randomly sheared into short fragments of approximately 350 bp. The obtained fragments were end-repaired, A-tailed, and further ligated with Illumina adapters. The adapter-ligated fragments were size-selected, PCR-amplified, and purified. The library was quantified using Qubit 3.0 (Invitrogen, USA) and real-time PCR, and the fragment size distribution was examined using Agilent 5400 Fragment Analyzer system (Agilent, USA). According to the effective library concentration and the required data volume, the quantified libraries were pooled and sequenced on an Illumina sequencing platform. To obtain high-quality clean reads for subsequent analysis, raw data were subjected to strict quality filtering using the following procedures: (1) Reads with more than 40% of low‑quality bases (quality score ≤ 20) were discarded; (2) Reads containing more than 10% ambiguous bases (N) were removed; (3) Reads with adapter contamination (overlap > 15 bp and ≤ 3 mismatches) were trimmed. Subsequently, the high-quality clean reads were de novo assembled into contigs using SPAdes v3.10.0 software [40].

### Genome collection and sequence quality assessment

We retrieved publicly available genomes annotated as *Staphylococcus epidermidis* from the RefSeq database (accessed by August 2025). Genomes derived from metagenome-assembled genomes and those belonging to large multi-isolate projects were excluded. In total, we identified 1,742 unique *S. epidermidis* BioSample entries. These, together with the 94 isolates sequenced in this study, yielded a final dataset of 1,836 genomes. To ensure correct species assignment, all genomes were compared against the reference genome of the *Staphylococcus epidermidis* ATCC 14,990 strain (assembly accession GCF_006094375.1_ASM609437v1) pairwise using FastANI v1.34, applying a ≥ 95% average nucleotide identity (ANI) threshold [41], genomes falling below this threshold were removed from the dataset. Using CheckM on the Galaxy platform (https://usegalaxy.org/) with default parameters to assess completeness and contamination for all genomes, including both RefSeq genomes and newly sequenced genomes. Only genomes with completeness ≥ 95% and contamination ≤ 5% were retained, and genomes failing these criteria were excluded from downstream analyses. To ensure no duplicate genomes remained in the analysis, we performed rigorous deduplication: (1) We used the duplicate detection and removal function in Panaroo, which automatically identifies and excludes identical or nearly identical genomes (such as multiple GCF assemblies corresponding to the same strain). (2) We further manually curated all strain names, culture collection identifiers, and accessions to remove redundant entries (e.g., ATCC 12228 / NCTC 13360 and their synonymous GenBank entries).

### Generating phylogenetic tree and clustering isolate into clonal clusters

The core genome for each isolate was identified using Panaroo (v1.5.2), a tool designed for pangenome analysis [42]. Panaroo performs multiple sequence alignment (MSA) for each core gene and automatically concatenates these alignments to generate a core genome alignment. Single nucleotide polymorphisms (SNPs) were subsequently extracted from the core gene alignment using snp-sites (https://sanger-pathogens.github.io/snp-sites/), resulting in a SNP-only multiple sequence alignment. This concatenated alignment was subsequently used to construct a phylogenetic tree using IQTREE (v3.0.1) with the Maximum Likelihood method. The best-fitting model for the data was determined by ModelFinder integrated in IQTREE, and the GTR + GAMMA model was selected [43]. Bootstrap values were calculated with 1,000 replicates to assess the robustness of the tree topology. We applied Fastbapsv.1.0.8, a Bayesian hierarchical clustering approach, to classify the genomes into clusters comprising genetically related individuals [44].

### Identification of ST, antimicrobial resistance, and virulence genes

Isolates were assigned to sequence types (STs) using MLST 2.23.0 (https://github.com/tseemann/mlst) to query the PubMLST database (https://pubmlst.org/organisms/staphylococcus-epidermidis). Antimicrobial resistance genes were annotated using ABRicate v1.0.1 with the CARD (Comprehensive Antibiotic Resistance Database) [45], and virulence genes were identified using ABRicate with the VFDB (Virulence Factor Database) [46]. We used the minimum thresholds of > 80% for sequence coverage and > 90% sequence identity for comparing query sequences from our data set with the curated sequences in the CARD database. Because *S. epidermidis* harbors relatively few virulence genes, a relaxed threshold of > 60% sequence coverage and > 70% sequence identity was applied to compare the query sequences from our dataset with the curated sequences in the VFDB database.

### Analysis of mutation, recombination, and genomic diversity

Core-genome alignments were generated using Parsnp v2.1.5 (https://github.com/marbl/parsnp) with the reference genome *S. epidermidis* (GCF 007672455.1 ASM767245v1) and the following parameters: -c --no-partition --min-ref-cov 0.9 --min-ani 95 -x -p 10. The resulting core-genome alignment was used as input for Mcorr (https://github.com/kussell-lab/mcorr), and 1,000 bootstrap replicates were performed with default settings to estimate recombination-related parameters [18, 19]. Mcorr employs a coalescent-based model to quantify the correlation of substitutions between loci separated by N bp and calculates evolutionary parameters, including diversity (*d*), mutational divergence (θ), recombinational divergence (ϕ), and recombination coverage (*c*). To evaluate the impact of uneven geographic sampling on Mcorr inference, we generated a country-balanced dataset by randomly down-sampling isolates from overrepresented countries without replacement so that each country contributed a comparable number of genomes (Table S3). The balanced dataset was then analyzed using the same Parsnp and Mcorr pipeline as described above.

### Analysis for open or close genomes

The pan-genome of *S. epidermidis* was constructed using the gene presence-absence matrix generated by Panaroo, which included all 1,836 genomes. The cumulative number of accessory genes as a function of sequenced genomes was then calculated using R package Panstripe v0.3.3 [47]. This analysis was also performed to each phylogroup, using the group-specific gene presence-absence matrix. Furthermore, the overall gene presence-absence matrix was analyzed using the Heaps’ law model in R to estimate the parameter ɑ, which was then used to assess genome openness based on the saturation curve in a conventional manner.

### Inference of gene gain and loss events

Gene gain and loss events were inferred using a phylogeny-based ancestral state reconstruction approach. A core genome phylogeny was reconstructed from a recombination-filtered alignment generated using Gubbins v3.4.3 [48], ensuring that branch lengths reflect clonal evolutionary divergence. Gene presence-absence data were extracted from the pan-genome matrix generated by Panaroo and converted into a binary matrix (presence = 1, absence = 0). For each gene, ancestral states across the phylogeny were inferred using a maximum likelihood approach implemented in the ace function from the R package ape, assuming an equal-rates (ER) model for state transitions. For each branch, transitions from absence to presence (0→1) were counted as gene gain events, and transitions from presence to absence (1→0) were counted as gene loss events. These events were summed across all genes to obtain the total number of gain and loss events per branch. The cumulative number of gene gain and loss events was calculated by summing events across all branches. The cumulative core genome branch length was calculated as the sum of all branch lengths in the phylogeny (expressed as substitutions per site).

### Homology analysis of *icaABCDR* operon

The *icaABCDR* sequences of various *Staphylococcus* strains were obtained from the RefSeq database. The protein sequence of the *icaABCDR* operon (SERP2292-6) from *S. epidermidis* RP62A (GCF_000011925.1) was used as a reference, and amino acid sequences were aligned using MAFFT v7.526 to calculate similarity indices. The phylogenetic tree was constructed from the concatenated, aligned amino acid sequences using IQ-TREE v3.0.1 with 1,000 ultrafast bootstrap replicates. Visualization of the phylogenetic tree and heatmaps was performed using the R v 4.5.1.

### Identification of genes enriched in blood-derived isolates

To identify genes associated with blood-derived *S. epidermidis* isolates, we performed a multivariable gene enrichment analysis based on the pangenome gene presence/absence matrix generated by Panaroo and isolate metadata (Table S2). The metadata, including isolation source, phylogroup, and country of origin, were obtained from the accompanying spreadsheet. Only isolates present in both datasets were retained for analysis. Isolates were classified as blood-derived or non-blood-derived according to their source metadata. Isolates annotated as blood, blood culture, peripheral blood, central venous pressure (CVP) line blood, blood culture from newborn, blood from newborn, blood from adult, bloodstream, or sepsis were defined as blood-derived and coded as 1; all other isolates were classified as non-blood-derived and coded as 0.

For each gene, presence was encoded as a binary variable, with 1 indicating presence and 0 indicating absence or missing data. To avoid unstable estimates caused by extremely rare or nearly ubiquitous genes, only genes with an overall frequency of > 5% and < 95% across all isolates were retained for downstream analyses. For each retained gene, a multivariable logistic regression model was fitted with isolation source (blood-derived vs. non-blood-derived) as the dependent variable, gene presence/absence as the main explanatory variable, and phylogroup and country as covariates. Odds ratios (ORs) were calculated by exponentiating the regression coefficients for the gene presence term.

### Statistical analysis

We carried out all visualization using the ggplot2 package in R v.4.5.1. Unless otherwise noted, default parameters were used for all programs. To identify genes associated with blood-derived *S. epidermidis* isolates, *P* values were adjusted using the Benjamini-Hochberg false discovery rate (FDR) method. Genes with an adjusted *P* value < 0.05 and OR > 1.5 were considered significantly enriched in blood-derived isolates.

## Supplementary Information

Supplementary Material 1.

Supplementary Material 2.

Supplementary Material 3.

Supplementary Material 4.

## Data Availability

The genome assemblies included in this analysis have been deposited in the Genome Sequence Archive (GSA) at the China National Center for Bioinformation under project PRJCA054181, Samples SAMC6319696-SAMC6319789. The repository can be accessed at https://ngdc.cncb.ac.cn/bioproject/browse/PRJCA054181. The corresponding BioProject accession numbers are listed in Table S2.
