# Supplementary material for: Global distribution, antimicrobial resistance, and virulence factors of Staphylococcus epidermidis revealed through population genomics
Source: BMC Genomics. 2026 May 14;27:598. doi: 10.1186/s12864-026-12922-5 (PMC13343668; doi:10.1186/s12864-026-12922-5)
Supplement: Supplementary file 4 — Supplementary Material 4. [file 12864_2026_12922_MOESM4_ESM.docx]

**Supplemental information**

**Global distribution, antimicrobial resistance, and virulence factors of *Staphylococcus epidermidis* revealed through population genomics**

Yan Yang, Jian-Hua Liu, Cong-Ran Li, Yuan-Biao Guo, Xue Li

Supplementary FIG S1. Mcorr-based comparison of evolutionary parameters across three phylogroups of *S. epidermidis* with country-number balanced sampling*.* Violin plots show the distributions of (A) overall nucleotide diversity (*d*), reflecting variation generated by both mutation and homologous recombination; (B) mutation-driven nucleotide diversity (θ); (C) recombination-associated diversity (ϕ); and (D) recombination coverage (c) across the three phylogroups.
